# Supplementary material for: Nurse-sensitive outcomes in district nursing care: A Delphi study
Source: PLoS One. 2021 May 13;16(5):e0251546. doi: 10.1371/journal.pone.0251546 (PMC8118269; doi:10.1371/journal.pone.0251546)
Supplement: S1 Appendix — (DOCX) [file pone.0251546.s001.docx]

**S1 Appendix: Overview of identified potential nurse-sensitive outcomes, corresponding definitions and references.**

**Identifying nurse-sensitive outcomes**

Potential nurse-sensitive outcomes for district nursing were identified using the following literature:

- Adams, C. E., Wilson, M., Haney, M., & Short, R. (1998). Using the outcome-based quality improvement model and OASIS to improve HMO patients' outcomes. Outcome Assessment and Information Set. *Home healthcare nurse*, *16*(6), 395-401.
- Akpan A, Roberts C, Bandeen-Roche K, Batty B, Bausewein C, Bell D, et al. Standard set of health outcome measures for older persons. BMC Geriatr. 2018;18(1).
- Bryan, S., Davis, J., Broesch, J., Doyle-Waters, M. M., Lewis, S., Mcgrail, K., ... & Sawatzky, R. (2014). Choosing your partner for the PROM: a review of evidence on patient-reported outcome measures for use in primary and community care. *Healthcare Policy*, *10*(2), 38.
- Bryant, L. L., Floersch, N., Richard, A. A., & Schlenker, R. E. (2004). Measuring healthcare outcomes to improve quality of care across post–acute care provider settings. *Journal of nursing care quality*, *19*(4), 368-376.
- Caminal, J., Starfield, B., Sánchez, E., Casanova, C., & Morales, M. (2004). The role of primary care in preventing ambulatory care sensitive conditions. *The European Journal of Public Health*, *14*(3), 246-251.
- Coster, S., Watkins, M., & Norman, I. J. (2018). What is the impact of professional nursing on patients’ outcomes globally? An overview of research evidence. *International journal of nursing studies*, *78*, 76-83.
- Department of Health and Human Services, Agency for Healthcare Research and Quality. 2008. “AHRQ Quality Indicators. Prevention Quality Indicators: Technical Specifications, Version 3.2” [accessed October 18, 2017]. Available at http://www.qualityindicators.ahrq.gov.
- HealthMeasures. PROMIS, Patient-Reported Outcomes Measurement Information System. [Online] Northwestern University, 2018. [accessed November 08, 2018]. Available at http://www.healthmeasures.net/explore-measurement-systems/promis .
- Hirdes, J. P., Fries, B. E., Morris, J. N., Ikegami, N., Zimmerman, D., Dalby, D. M., ... & Jones, R. (2004). Home care quality indicators (HCQIs) based on the MDS-HC. *The Gerontologist*, *44*(5), 665-679.
- Keleher, H., Parker, R., Abdulwadud, O., & Francis, K. (2009). Systematic review of the effectiveness of primary care nursing. *International journal of nursing practice*, *15*(1), 16-24
- Martin KS. The Omaha System: A key to practice, documentation, and information management. WB Saunders Co; 2004 Dec 1.
- Meadows, K. A. (2011). Patient-reported outcome measures: an overview. *British journal of community nursing*, *16*(3), 146-151.Meadows KA1.
- Moorhead, S., Johnson, M., Maas, M. L., & Swanson, E. (2018). *Nursing Outcomes Classification (NOC)-E-Book: Measurement of Health Outcomes*. Elsevier Health Sciences.
- Morris, J. N., Fries, B. E., Frijters, D., Hirdes, J. P., & Steel, R. K. (2013). interRAI home care quality indicators. *BMC geriatrics*, *13*(1), 127.
- Nakrem, S., Vinsnes, A. G., Harkless, G. E., Paulsen, B., & Seim, A. (2009). Nursing sensitive quality indicators for nursing home care: international review of literature, policy and practice.*International journal of nursing studies*, *46*(6), 848-857.
- Recio-Saucedo: Recio‐Saucedo, A., Dall'Ora, C., Maruotti, A., Ball, J., Briggs, J., Meredith, P., ... & Griffiths, P. (2017). What impact does nursing care left undone have on patient outcomes? Review of the literature.*Journal of Clinical Nursing*
- Russell, D., Rosati, R. J., Rosenfeld, P., & Marren, J. M. (2011). Continuity in home health care: is consistency in nursing personnel associated with better patient outcomes?. *Journal for healthcare quality*, *33*(6), 33-39.
- Shaughnessy, P. W., Hittle, D. F., Crisler, K. S., Powell, M. C., Richard, A. A., Kramer, A. M., ... & Mulvey‐Lawlor, K. L. (2002). Improving Patient Outcomes of Home Health Care: Findings from Two Demonstration Trials of Outcome‐Based Quality Improvement. *Journal of the American Geriatrics Society*, *50*(8), 1354-1364.

**Defining nurse-sensitive outcomes**

Different references were used for defining the outcomes. For most outcomes, multiple references were combined to one definition. Because all experts were from the Netherlands, mostly Dutch literature has been used.

- Akpan A, Roberts C, Bandeen-Roche K, Batty B, Bausewein C, Bell D, et al. Standard set of health outcome measures for older persons. BMC Geriatr. 2018;18: 36.
- Bakker, A. J. E. M., Habes, V., & Quist, G. (2016). *Klinisch redeneren bij ouderen: functiebehoud in levensloopperspectief*. Bohn Stafleu van Loghum.
- Beers MH, editor. Merck manual medisch handboek. Bohn Stafleu van Loghum; 2016 Jan 13.
- Gordon, M. (2014). *Handleiding verpleegkundige diagnostiek*. (4th. ed.). Amsterdam, the Netherlands: Reed Business Education.
- Herdman, T. H. (2014). *NANDA International Verpleegkundige diagnoses en classificaties 2012–2014*. Houten, the Netherlands: Bohn Stafleu van Loghum.
- Herdman, T. H., & Kamitsuru, S. (2014). *NANDA International, Inc., Nursing Diagnoses: Definitions & Classification 2015–2017*, (10th ed.). West Sussex, UK: John Wiley & Sons.
- Martin, K. S., & Scheet, N. J. (2005). The OMAHA system. *Applications for Community health nursing*, 1992.
- Moorhead, S., Johnson, M., Maas, M. L., & Swanson, E. (2018). *Nursing Outcomes Classification (NOC)-E-Book: Measurement of Health Outcomes*. Elsevier Health Sciences.
- Nederlandse Zorgautoriteit (NZa, Dutch healthcare authority). (2015). *Handboek Gebruik Zorgactiviteiten*. DBC Onderhoud.
- van Achterberg, T., Bours, G. J. J. W., & Eliens, A. M. (2011). *Effectief Verplegen 2* (3rd ed.). Dwingeloo, the Netherlands: Kavanah.
- van Achterberg, T., Bours, G. J. J. W., & Eliens, A. M. (2012). *Effectief Verplegen 1* (4th ed.). Dwingeloo, the Netherlands: Kavanah.
- World Health Organization. Lexicon of alcohol and drug terms published by the World Health Organization [internet]. Available via https://www.who.int/substance_abuse/terminology/who_lexicon/en/

Definitions previous identified and defined by van den Bulck et al. were often used with permission by the first author.

- van den Bulck AO, Metzelthin SF, Elissen AM, Stadlander MC, Stam JE, Wallinga G, Ruwaard D. Which client characteristics predict home‐care needs? Results of a survey study among Dutch home‐care nurses. Health & Social Care in the Community. 2019 Jan;27(1):93-104.

Newly added outcomes after round 1 were defined by the experts and checked by researchers from the research group (JDV, NB, MJS).

| **Outcome** | **Definition** | **Source** |
| --- | --- | --- |
| **Functional health** |  |  |
| Activities of daily living (ADL) | The extent to which the patient (together with the people around the patient) is independent in carrying out activities of daily living (ADL) such as washing / showering, external care, dressing and undressing, eating, and visiting the toilet. | van den Bulck |
| Frailty | The extent to which the patient is frail, whereby frailty is defined as a process of accumulating physical, psychological and/or social deficits in functioning that increases the chance of negative health outcomes. Frailty is characterized by the weak position that the patient has in society and/or the risk that the patient runs of not catching up with society, getting into social isolation or experiencing deterioration in terms of physical, mental or social functioning. | Bakker |
| Instrumental activities of daily living (IADL) | The extent to which the patient (together with the people around the patient) is independent in carrying out instrumental activities of daily living (IADL) such as housework, shopping, preparing meals, and making telephone calls. | van den Bulck |
| Mobility | The ability to move purposefully in one's own environment (indoors and outdoors), possibly with the help of (walking) aids. Think of climbing stairs, moving from a standing position to a sitting position, mobility in and around the bed, moving in or out of a bath/shower; movements in or out of the car, movements on foot, by bicycle or public transport. | van den Bulck; Moorhead |
| **Psysiologic health including neurocognitive health** |  |  |
| Bladder continence | The extent to which the patient has control over the excretion of urine. | van den Bulck |
| Bowel continence | The extent to which the patient has control over the excretion of faeces. | van den Bulck |
| Cognitive functioning | The extent to which the patient is able to record, process, reproduce and apply information based on his cognitive functions, such as intelligence, memory, attention and concentration, orientation ability, language and communication, decision making, and problem solving ability. | van den Bulck |
| Communication | The extent to which the patient is able to communicate effectively by being able to receive, interpret and express spoken, written or non-verbal messages. This also concerns the extent to which the patient has the skills to perform this (such as eye contact, speaking, articulating thoughts, forms of sentences and words, selective attention, and using body language and facial expressions). | van den Bulck; Herdman; Moorhead |
| Decision making | The extent to which the patient is able to make decisions regarding the provision of care, by making an assessment and choosing between two or more alternatives. | Herdman; Moorhead |
| Decubitus (Pressure ulcers) | The presence of decubitus, where decubitus is defined as damage to the skin and tissues under the skin as a result of local action of pressure or shear forces. | Bakker, Herdman |
| Dehydration | The presence of dehydration in the patient, where dehydration is defined as a condition in which there is a lack of bodily fluid. There is an unbalanced fluid balance and composition of the patient's body fluids, characterized by a relative lack of fluid in the body, which is not sufficient to meet the physiological needs. | van den Bulck; Bakker |
| Delirium | The presence of delirium in the patient, where delirium is defined as a reversible disorder in consciousness and cognition that develops within a short period of time. | Moorhead |
| Dyspnoea | The degree to which the patient experiences dyspnoea, where dyspnoea is defined as a situation where the balance between oxygen uptake and carbon dioxide release in the lungs is disturbed, which is accompanied by a feeling of shortness/lack of breath. | Achterberg |
| Fatigue | The extent to which the patient experiences long-term general fatigue, which leads to reduced capacity for physical and mental exertion at the usual level. | Moorhead |
| Fracture and wounds other than decubitus | The presence of new fractures and injuries, where injuries are defined as injuries to the skin (for example, damaged epidermis and / or dermis, such as skin tears, cuts or wounds from burns). Note: decubitus is included as a separate outcome. | Herdman |
| Infection | The presence of infections caused by bacteria, virus or parasite, regardless of the location of the inflammation. For example: urinary tract infection, respiratory tract infection, pneumonia, wound infection. | Beers |
| Multimorbidity | The presence of multimorbidity, defined as the presence of more than one (chronic) disease in the patient at the same time. | van den Bulck |
| Pain | The extent to which the patient experiences pain, where pain is defined as an unpleasant, sensory, and emotional experience, which can be subjective, continuous/recurrent, and sudden/slow-induced, caused by actual/imminent tissue damage, with every possible intensity (from mild to severe). | van den Bulck; Bakker |
| Polypharmacy | The presence of polypharmacy, defined as the chronic use of five or more medications at the same time. | Bakker |
| Unintentional weight loss | The presence of unintended weight loss in the patient, where unintended weight loss is defined as a weight loss of more than 10% in the last six months or more than 5% in the last month. | Bakker |
| **Psychosocial health** |  |  |
| Anxiety | The extent to which the patient experiences a feeling of unease or insecurity with a source that is usually unclear or unknown to the patient. | van den Bulck |
| Loneliness | The extent to which the patient experiences loneliness, whereby loneliness is defined as the subjective experience of an unpleasant or unacceptable lack of (quality of) certain relationships. This may involve emotional loneliness (lack of an emotionally close bond and/or intimate relationship) or social loneliness (lack of meaningful relationship with a wide circle of people). | Bakker |
| Participation in social activities | The extent to which the patient participates in society in a way that is meaningful to the patient, such as (un)paid work, following education, and participation in sports activities and other leisure activities. | van den Bulck |
| Signs of depression | The extent to which the patient experiences periods of reduced, (seriously) depressed mood, characterized by, among other things, loss of interest or pleasure in activities, less energy, insomnia, and reduced self-esteem and self-confidence. | van den Bulck |
| **Health knowledge and behaviour** |  |  |
| Autonomy | The extent to which the patient has control over his own life in various areas of life (such as living, working and social contacts) and any support therein. | van den Bulck |
| Compliance | The extent to which the behaviour of a patient matches the established therapy or the health promotion plan. | Herdman |
| Falls | The presence of fall incidents, where a fall incident is defined as an unintended change of body position that results in a fall on the ground or another lower level. | van den Bulck; Bakker |
| Knowledge of the patient | The ability of the patient to remember and interpret information. | Martin |
| Problem behaviour | The extent to which the patient exhibits behaviour that has or may have a negative impact on his own health, well-being and/or (the relationship with) other people such as verbal or physical violence, distrust or hallucinations, compulsions or astray. | van den Bulck |
| Substance use | The extent to which the patient absorbs psychoactive substances in a harmful or dangerous way, including alcohol and (illegal) drugs. | World Health Organization |
| **Perceived health** |  |  |
| Quality of life | The extent to which the patient values his or her quality of life, whereby quality of life is defined as a positive experience of one's own current living conditions. | Moorhead |
| Satisfaction with district nursing care | The extent to which the patient is satisfied with the care provided by district nursing care. |  |
| Meaningful life | Living from what is really important to a person. | Experts |
| **Family health** |  |  |
| Informal caregiver burden | The extent to which the informal caregiver of the patient experiences a balance in burden/vulnerabilities (load) and the resources of the caregiver to carry the burden (capacity). | van den Bulck |
| **Death** |  |  |
| Death | The patient has died. | NZa |
| Place of death | The patient has died at the desired place of death. | Akpan |
| Quality of dying and death | Discuss timely the options and take care of counselling in the palliative and terminal phase. | Experts |
| **Healthcare consumption** |  |  |
| Duration of district nursing | Total duration that a patient receives district nursing care (e.g. in weeks). | NZa; Experts |
| Emergency department or service use | The patient makes use of the emergency department or emergency service (out of office general practitioner visit). | NZa |
| General practitioner visit | The patient has visited the doctor or the doctor has visited the patient at home during office hours. | NZa |
| Intensity of district nursing | Total number of minutes of care per week that a patient receives district nursing care. | NZa; Experts |
| Nursing home admission | The patient has an admission to a nursing home with no prospect of returning home (no first-line residence or rehabilitation). | NZa |
| Planned hospital admission | The patient has been scheduled to be admitted or treated at the hospital. The patient has stayed in the hospital for at least one night. | NZa; ICHOM |
| Total time at home | Total time that a patient lives independently at home (e.g. in months or days per year). | Experts |
| Unplanned hospital admission | The patient has been admitted to hospital or treated at the hospital unplanned. The patient has stayed in the hospital for at least one night. | NZa; Akpan |
| Unplanned hospital readmission | Within three months of a previous hospital visit, the patient has been admitted to hospital or treated unplanned. The patient has stayed in the hospital for at least one night. | NZa |
