# Supplementary material for: Nurse-sensitive outcomes in district nursing care: A Delphi study
Source: PLoS One. 2021 May 13;16(5):e0251546. doi: 10.1371/journal.pone.0251546 (PMC8118269; doi:10.1371/journal.pone.0251546)
Supplement: S2 Appendix — (DOCX) [file pone.0251546.s002.docx]

**S2 Appendix: Examples of questionnaire questions round one and round two**

**Round one example question on relevance and influenceability of mobility as an outcome**

Please fill in how relevant you think this outcome is as a measurement for the quality of district nursing care

Please fill in how influenceable you think this outcome is by your work in a district nursing team.

**Mobility**

Definition: The ability to move purposefully in one's own environment (indoors and outdoors), possibly with the help of (walking) aids. Think of climbing stairs, moving from a standing position to a sitting position, mobility in and around the bed, moving in or out of a bath / shower; movements in or out of the car, movements on foot, by bicycle or public transport.

| Completely  **NOT relevant** |  |  |  |  |  |  |  | Completely **relevant** | Completely  **NOT influenceable** |  |  |  |  |  |  |  | Completely **influenceable** |
| --- | --- | --- | --- | --- | --- | --- | --- | --- | --- | --- | --- | --- | --- | --- | --- | --- | --- |
| **1** | **2** | **3** | **4** | **5** | **6** | **7** | **8** | **9** | **1** | **2** | **3** | **4** | **5** | **6** | **7** | **8** | **9** |
| ⧠ | ⧠ | ⧠ | ⧠ | ⧠ | ⧠ | ⧠ | ⧠ | ⧠ | ⧠ | ⧠ | ⧠ | ⧠ | ⧠ | ⧠ | ⧠ | ⧠ | ⧠ |

**Round two example question on relevance and influenceability of mobility as an outcome**

Please fill in how relevant you think this outcome is as a measurement for the quality of district nursing care.

| **Mobility** | | | | | | | | | |
| --- | --- | --- | --- | --- | --- | --- | --- | --- | --- |
| Individual score | | | | | | | |  | |
| Median (group score) | | | | | | | | 7 | |
| Disagreement Index (Score <1 = agreement) | | | | | | | | 0,37 | |
| Completely  **Not relevant** | |  |  | Neutral |  |  |  | | Completely **relevant** |
| **1** | **2** | **3** | **4** | **5** | **6** | **7** | **8** | | **9** |
| ⧠ | ⧠ | ⧠ | ⧠ | ⧠ | ⧠ | ⧠ | ⧠ | | ⧠ |

Please fill in how influenceable you think this outcome is with your work in a district nursing team.

| **Mobility** | | | | | | | | | |
| --- | --- | --- | --- | --- | --- | --- | --- | --- | --- |
| Individual score | | | | | | | |  | |
| Median (group score) | | | | | | | | 6 | |
| Disagreement Index (Score <1 = agreement) | | | | | | | | 0,22 | |
| Completely  **Not influenceable** | |  |  | Neutral |  |  |  | | Completely **influenceable** |
| **1** | **2** | **3** | **4** | **5** | **6** | **7** | **8** | | **9** |
| ⧠ | ⧠ | ⧠ | ⧠ | ⧠ | ⧠ | ⧠ | ⧠ | | ⧠ |
