# Supplementary material for: Nurse-sensitive outcomes in district nursing care: A Delphi study
Source: PLoS One. 2021 May 13;16(5):e0251546. doi: 10.1371/journal.pone.0251546 (PMC8118269; doi:10.1371/journal.pone.0251546)
Supplement: S3 Appendix — (DOCX) [file pone.0251546.s003.docx]

**S3 Appendix: Equation to calculate disagreement index (DI)**

| Lower Limit IPR = 30^th^ percentile of the series of ratings  Upper Limit IPR = 70^th^ percentile of the series of ratings  IPR = (Upper Limit IPR) – (Lower Limit IPR)  IPRCP (Central Point of IPR) = Average of Upper Limit IPR and Lower Limit IPR  Asymmetry Index = 5^*^ – (IPRCP)  IPRAS = 2.35^*^ + (1.5^*^ ∙ Asymmetry Index)  Disagreement Index (DI) = IPR/IPRAS |
| --- |

*Notes: IPR=Interpercentile Range; IPRCP=interpercentile Range Central Point; IPRAS = Interpercentile Range Adjusted for Symmetry. *Numbers determined by RAND/UCLA Appropriateness Method (16)*
